# Supplementary material for: Delayed Recovery in Idiopathic Sudden Sensorineural Hearing Loss
Source: J Clin Med. 2022 May 16;11(10):2792. doi: 10.3390/jcm11102792 (PMC9143329; doi:10.3390/jcm11102792)
Supplement: Supplementary file 1 [file jcm-11-02792-s001.zip › jcm-1674033-supplementary.pdf]

Supplementary Table S1.

	Grade 1	Grade 2	Grade 3	Grade 4
Immediately after treatment				
Number of patients	57	19	22	32
Ipsilateral PTA (dB HL)*	19.1 ± 11.7	32.4 ± 8.41	69.9 ± 18.6	63.9 ± 26.3
Contralateral PTA (dB HL)†	15.3 ± 11.9	11.1 ± 6.21	18 ± 12.3	16.5 ± 10.8
Interaural difference (dB HL)‡	3.82 ± 3.65	21.4 ± 7.07	51.9 ± 22.7	47.4 ± 24.1
Two months after treatment completion				
Number of patients	60	23	22	25
Ipsilateral PTA (dB HL)*	19.2 ± 12	34.5 ± 10.3	71.6 ± 15.8	57.8 ± 19.8
Contralateral PTA (dB HL)†	15.7 ± 12.3	11.8 ± 5.77	24 ± 13.6	13.5 ± 9.7
Interaural difference (dB HL)‡	3.57 ± 3.32	22.7 ± 9.45	47.7 ± 22.8	44.3 ± 21.6
P value	0.96*	0.48*	0.74*	0.33*
	0.87†	0.68†	0.13†	0.27†
	0.76‡	0.62‡	0.54‡	0.60‡

*Unpaired t-test was performed to compare ipsilateral pure-tone average (PTA) between the immediately after treatment and the 2 months after treatment.

†Unpaired t-test was performed to compare contralateral PTA between the immediately after treatment and the 2 months after treatment.

‡Unpaired t-test was performed to compare interaural difference between the immediately after treatment and the 2 months after treatment.
